# Novel Approaches in Biomolecule Labeling

**DOI:** 10.3390/biom11121809

**Published:** 2021-12-02

**Authors:** Cyrille Sabot, Péter Kele

**Affiliations:** 1 Normandie University, CNRS, UNIROUEN, INSA Rouen, COBRA, 76000 Rouen, France; 2 Chemical Biology Research Group, Institute of Organic Chemistry, ELKH Research Centre for Natural Sciences, Magyar tudósok krt 2, H-1117 Budapest, Hungary

The selective functionalization of biomolecules such as proteins, nucleic acids, lipids or carbohydrates is a focus of persistent interest due to their widespread use, ranging from basic chemical biology research to gain insight into biological processes to the most promising biomedical applications, including the development of diagnostics or targeted therapies. Although specific functionalization can be achieved via genetic engineering techniques such as the fusion of fluorescent proteins or self-labelling tags (SNAP-tag, CLIP-tag, HaloTag), chemical conjugation alternatives overcome some of their limitations while providing new, exciting perspectives.

Chemical ligation can be divided into two distinct classes of reaction: (a) bioconjugation, based on the direct modification of native biomolecules, such as amino acid side-chains of proteins, which are intrinsically nucleophilic (mainly Cys, Lys); (b) bioorthogonal chemistry, which requires the prior metabolic or genetic installation of a biologically inert functional group into a biomolecule or biosystem. This chemical reporter is subsequently detected with a complementary reactive group bearing a fluorophore or an affinity handle. Bioorthogonal reactions can advantageously take place inside living systems, whereas bioconjugation reactions are mostly limited to in vitro applications.

The choice of a chemical conjugation strategy has mainly been driven by its ability to proceed rapidly, chemoselectivity, and with high yield under physiological conditions to meet the requirements of biomolecules. However, with recent developments, many other attractive features can be considered when selecting a reaction. For example, ultrafluorogenic ligation tools have dramatically improved the signal-to-noise ratio, allowing live cell labeling experiments under no-wash conditions. In addition, photoactivable conjugations enable the effective identification of weak, transient biomolecular interactions, or provide an unprecedented level of spatiotemporal control of biological processes [1]. Sophisticated conjugation technologies or platforms with exceptional modularity are also available for the dual labeling of proteins [2] or for the engineering of antibody–drug conjugates [3]. This Special Issue of *Biomolecules,* devoted to “Novel Approaches in Biomolecule Labeling”, provides detailed studies illustrating the potential of biocompatible ligation strategies through a variety of applications.

The site-specific introduction of a bioorthogonal reporter into biomolecules such as proteins or antibodies in the context of targeted delivery provides an anchoring site for the subsequent easy attachment of any cargo of interest, such as pharmacophores. For example, Tsao et al. [4] reported the fluorogenic site-specific incorporation of an alkyne functional group onto a protein of interest, namely maltose-binding protein (MBP), fused to a small helical peptide tag (dC10a tag) presenting two Cys residues capable of specifically reacting with novel dimaleimide-based conjugating linkers. These linkers consist of a coumarin platform, functionalized at each side by an alkyne group, for subsequent cargo loading, and two maleimide units ensuring both bioconjugation and quenching of the coumarin’s fluorescence [5]. Accordingly, the conjugation of the linker to the protein can be advantageously monitored by fluorescence under “no wash” conditions.

While, in the latter example, the fluorescence emission was triggered by a bioconjugation reaction involving the consecutive addition of two Cys residues, Németh et al. [6] reported the design of novel bioorthogonally activatable fluorogenic probes. Such probes, also termed bioorthogonal smart probes [7], are particularly interesting for the labeling of specific proteins in biological systems while minimizing background fluorescence from an unreacted probe. However, most notable examples of fluorescence turn-on probes are UV/blue-excitable, whose low emission wavelengths can interfere with the autofluorescence of biological samples. This study addresses the lack of robust fluorogenic probes that emit light in the orange, red region by creating a fluorogenic dye possessing a large Stokes-shift. This was achieved by designing a coumarin dye with an extended π-electron system due to a vinylpyridinium moiety at C3 position, and a two-in-one bioorthogonal and fluorescence quencher phenyltetrazine handle at position C4. Noticeably, this probe was found to be suitable for stimulated emission depletion (STED) super-resolution microscopy.

In the context of medical diagnostic imaging, the radiolabeling of biomolecules constitutes an important alternative to the aforementioned optical imaging approaches, by providing deeper tissue penetrations. Metallic radionucleides require the use of bifunctional chelators, which consist of a metal sequestration unit and a conjugatable function for covalent attachment to the biomolecules of interest. Different bioconjugation strategies have been developed, whose choice is guided by the preservation of biomolecules activity, in vivo behavior (thermodynamic, kinetic, stability, etc.), and synthetic accessibility. While the challenge of large molecules primarily lies in generating homogeneous bioconjugates, the difficulty with small molecules is associated with the preservation of their biological activity. In this context, Poret et al. [8] developed two original ^111^In radiolabeled cyclic oligopeptide ligands (urotensin II, and urantide) targeting the urotensin receptor (UT), which is highly expressed in several types of solid tumors including lung, prostate, and breast. Importantly, both these radiolabeled urotensinergic ligands exhibited similar binding affinities to the native peptides and activity, demonstrating successful bioconjugation.

Flon and co-workers [9] convince us that there is still room for further improvements in CuAAC-based click-labeling schemes. Their smartly designed probe combines the rate-accelerating effects of bidentate azides with the fluorescent properties of copper-binding azaphtalimide frames. The result is an unprecedented approach where the novel azidoethyl-azaphtalimide probe serves as the fluorescent label and the Cu(I)-complexing ligand at the same time. This all-in-one chelating fluorescent azide probe facilitated the reaction rate compared to conventional Cu(I)-chelating ligands used in CuAAc schemes and its applicability was demonstrated in the fluorescent labeling schemes of short alkyne modified peptides in vitro and on alkyne-modified cells, in cellulo.

UV-light based methods applied on live cells are often condemned for the cytotoxicity of high-energy irradiation. Pull-down assays aiming to track down protein–protein interactions, however, benefited greatly from UV-activatable crosslinking probes capable of capturing even weakly binding partners. In his paper, J-E. Hoffmann [10] provides a focused review on a small set of genetically encodable, bifunctional non-canonic amino acids (ncAAs). On the one hand, such ncAAs are site-specifically incorporated into proteins of interests, resulting in a precise installation of a minimally perturbing photo-activatable cross-linker handle. Upon UV-irradiation, any interaction partners in the close proximity of the tagged protein are captured by covalent bond formation. On the other hand, the bifunctional ncAAs also bear a function that allows for further highly specific modulation of the POI carrying its interacting partner, e.g., via click-chemistry. This second modification could result in the installation of a wide range of functionalities, from fluorescent labels to pull-down handles. The reviewed ncAAs demonstrate that having a clickable group in each crosslinked assemblies of proteins could greatly facilitate the generation of a wide variety of interactome fingerprints as a result of a combination of different codon and cell states.

The work of Krell and Wagenknecht [11] also sheds (UV) light on the beneficial effects of a short burst of higher energy irradiation in the context of fluorogenic modification of RNA using tetrazole-photoclick chemistry. Direct attachment of a bromoaryltetrazole moiety to uracil furnished the conventional diaryltetrazole, which undergoes UV-B induced bioorthogonal photoclick reactions with various olefins. Their bromoaryltetrazole-modified uridine building block was readily incorporated via solid-phase synthesis into two RNA strands, either at an internal or a terminal position. While such photoclick reactions of tetrazoles are inherently fluorogenic, the resulting diarylpyrazolines are usually weakly fluorescent. By reading on the manuscript, we can find out how the fluorescent signal can be boosted. Maleimide–dye conjugates are covalently attached to the tetrazole modified RNA strands through photoclick reaction of the bromoaryltetrazole-uracil and the maleimide. Efficient energy transfer between the pyrazoline product and the appending strong red emitter dyes results in a ca. 10-fold fluorescence increase in the red channel. Such photo-click-reaction-based methods with enhanced red fluorogenicity and excellent spatiotemporal resolution may bring live cell RNA imaging to a new level.
